# Accuracy of sonographic fetal weight estimation in full-term singleton pregnant women

**DOI:** 10.12669/pjms.35.1.373

**Published:** 2019

**Authors:** Emre Erdem Tas, Edip Alptug Kir, Gamze Yilmaz, Ayse Filiz Yavuz

**Affiliations:** 1 Emre Erdem Tas, Assistant Professor, Department of Obstetrics and Gynecology, Ankara Yildirim Beyazit University, Faculty of Medicine, Ankara, Turkey; 2 Edip Alptug Kir, M.D. Department of Obstetrics and Gynecology, Ankara Yildirim Beyazit University, Faculty of Medicine, Ankara, Turkey; 3 Gamze Yilmaz, M.D. Department of Obstetrics and Gynecology, Ankara Ataturk Training and Research Hospital, Ankara, Turkey; 4 Prof. Ayse Filiz Yavuz, Department of Obstetrics and Gynecology, Ankara Yildirim Beyazit University, Faculty of Medicine, Ankara, Turkey

**Keywords:** Prenatal ultrasonography, fetal weight, Birth weight, Pregnancy

## Abstract

**Objectives:**

To investigate the factors which might influence the sonographic fetal weight estimation (SFWE) accuracy.

**Methods:**

This prospective study was conducted among 949 singleton term pregnant women who delivered at a tertiary center, from January 2017 to December 2017. All participants’ maternal (i.e. parity, age, body mass index and gestational weight gain during pregnancy), fetal sonographic (i.e. fetal presentation, amniotic fluid index, localization of placenta and estimated fetal weight) and neonatal (birth weight and gender) characteristics were recorded. A p<0.05 was considered significant.

**Results:**

The mean absolute percent error (APE) values of SFWE was 8.2±6.5 percent, and overall failure ratio (APE >10%) was 33%. In failure group, primiparous woman and cephalic presentation fetus were significantly more common compared to accuracy group (55.9% *vs*.44.8%; *p*=0.001 and 98% *vs*. 95.2%; *p*=0.03, respectively). In contrast, the mean neonatal birth weight (NBW) value was significantly lower in failure group compared to success group (3250±565 gr *vs*. 3404±410 gr; *p*=0.001). The correlation between SFWE and NBW was linear, however negative, and significant (*p*=0.001). Logistic regression analysis revealed that primiparous woman, cephalic presentation fetus and <3300 gr NBW were independent risk factors for the SFWE failure (relative risks were 1.6, 2.8 and 2.4 respectively, p<0.05).

**Conclusion:**

SFWE has a high correlation with NBW, however it’s accuracy is still unsatisfactory, and depend on many unpredictable and inconsistent factors.

## INTRODUCTION

Birth weight is an important factor determining maternal and neonatal well-being that significantly influences obstetric management.^1^ Hence, fetal weight estimation has become an important aspect of examinations in the late period of pregnancy. To this end, various techniques, including clinical examinations, such as Leopold’s maneuvers^2^, sonography^3^, and magnetic resonance imaging^4^, have been used. However, sonography has become the most common method for estimating fetal weight worldwide, because of its objectivity and ease of use.

It is still debated whether sonography is a reliable tool for estimating fetal weight accurately.^5^,^6^ In previous studies,^4^,^7^,^8^ the accuracy of sonographic fetal weight estimation (SFWE), which is regarded by many as a determinant of fetal weight within a 10.0% error of neonatal birth weight (NBW), has varied widely.^9^,^10^ Moreover, in a quantitative review^8^, the mean accuracy rate of SFWE was reported to be 56.0% in full-term pregnant women. Evaluating the validity of sonography, previous studies^8^,^11^-^19^ have also evaluated whether maternal, fetal sonographic, neonatal, and technical characteristics play a role in determining the accuracy of SFWE. However, the results have been inconsistent and it is not yet known which factor(s) increase prediction failure.^5^ Therefore, the aim of this study was to investigate the accuracy, as well as factors affecting the accuracy, of SFWE in full-term uncomplicated singleton pregnant women.

## METHODS

We conducted a prospective cohort study of full-term singleton pregnant women who were admitted to the Obstetrics and Gynecology Unit of a university hospital between January and December 2017. All participants provided informed written consent. This study was approved by the Ethical Review Board Committee of our institution (approval no.: 26379996/125). Research was conducted in accordance with the World Medical Association Declaration of Helsinki, revised in 2000, Edinburgh.

Patients with congenital malformations of the fetus, placental abnormalities, maternal medical conditions, or pregnancies complicated by stillbirths were excluded from the study to ensure objectivity of the data. In the study group, maternal (age, parity [primiparity *vs*. multiparity], gestational age [weeks], height [cm], antepartum weight [kg], weight gain during pregnancy [kg], body mass index, and stage of labor at sonographic examination [active phase *vs*. latent phase]), fetal sonographic (SFWE [g], amniotic fluid volume [mm], placental localization [anterior *vs*. other]), and neonatal (NBW [g] and sex) characteristics of the participants were recorded during the study period. Body mass index (kg/m^2^) was calculated as the antepartum weight in kilograms divided by the height in meters squared.

The sonographic examinations were performed using Esaote My Lab 60 (Esaote, Genova, Italy) and a 3.5-MHz curvilinear probe. Fetal measurements included biparietal diameter, abdominal circumference, and femur length. For each patient, the measurements were repeated three times and the average values were recorded for analysis. Thereafter, SFWE was calculated using Hadlock’s formula: Log_10_ birth weight = 1.335 – (0.0034 × abdominal circumference × femur length) + (0.0316 × biparietal diameter) + (0.0457 × abdominal circumference) + (0.1623 × femur length).^20^ Additionally, amniotic fluid volume, which was measured in all four quadrants, and placental localization were recorded.

Training practitioners have been associated with increased SFWE accuracy and prolonged time intervals between examination and delivery have been associated with reduced SFWE accuracy.^12^,^13^ Therefore, all sonographic examinations were performed by two experienced obstetricians (E.E.T. and A.F.Y.) during hospital admission. If delivery did not occur within one week of admission, the sonographic examinations were repeated by the same obstetricians. Concurrent to the sonographic examinations, vaginal examinations were performed by the same obstetricians. Patients were classified according to the stage of labor: active (cervical dilation ≥4.0 cm) or latent (cervical dilation <4.0 cm) phase.

The main standard for comparing SFWE was NBW, which is measured after birth by midwives using a digital scale. SFWE accuracy was analyzed using the percentage error ([estimated weight – actual weight] × 100/actual weight), although the absolute percentage error (APE) was used for the statistical analysis. If the APE fell within the 10.0% range, SFWE was considered a success; if not, SFWE was considered a failure. Patients were grouped according to SFWE accuracy (i.e., success *vs*. failure) and compared in relation to the investigated parameters to determine factors associated with SFWE failure.

### Statistical Analyses

Descriptive parameters were expressed as the mean and standard deviation (continuous variables) and as numbers and percentages (categorical variables). Independent samples *t*-tests and Chi-square tests were used to analyze the data and compare the groups (i.e., success *vs*. failure). The relationship between NBW and the APE of SFWE accuracy was evaluated using Pearson’s correlation coefficient. Thereafter, receiver operating characteristic curve analysis was used to determine cutoff values of NBW for predicting an increased risk of SFWE failure. Variables with a *p* < 0.05 were included in the binary logistic regression analysis and the influence of each factor on the accuracy of SFWE was evaluated. Statistical analyses were conducted using Statistical Package for the Social Sciences for Windows (software version 21.0; IBM Corp., Armonk, NY, USA). A *p* < 0.05 was considered statistically significant. Relative Risk (RR) and 95.0% Confidence Intervals (CIs) were calculated.

## RESULTS

During the study period, 949 (74.3%) of the 1,278 women with full-term singleton pregnancies fulfilled the inclusion criteria and were enrolled into the study. In the study group, the mean absolute error and mean APE was 269 ± 212 g and 8.2 ± 6.5%, respectively. The APE was >10.0% in 313 patients. The overall failure rate was 33.0%. The maternal, fetal sonographic, and neonatal characteristics of the patients are summarized in Table-I.

**Table-I:** Maternal, fetal sonographic, and neonatal characteristics of the patients

Characteristic	Patients (*n* = 949)
*Maternal*	
Age (years), mean ± SD	28.1 ± 5.4
*Parity, n (%)*	
Primiparity	460 (48.4)
Multiparity	489 (51.6)
Gestational age (weeks), mean ± SD	39.1 ± 1.3
Maternal height (cm), mean ± SD	161.7 ± 5.3
Maternal pregestational weight (kg), mean ± SD	65.0 ± 12.4
BMI (kg/m^2^), mean ± SD (range)	29.2 ± 4.8
Weight gain during pregnancy (kg), mean ± SD	11.3 ± 4.5
*Labor stage, n (%)*	
Active phase	114 (12.0)
Latent phase	835 (88.0)
Fetal sonographic	
Estimated fetal weight (g), mean ± SD	3,430 ± 360
Amniotic fluid volume (mL), mean ± SD	104 ± 34
*Placental localization, n (%)*	
Anterior	286 (30.1)
Other (posterior, fundal, or lateral)	663 (69.9)
*Fetal presentation, n (%)*	
Cephalic	913 (96.2)
Non-cephalic (breech or transverse)	36 (3.8)
Neonatal	
*Sex, n (%)*	
Male	514 (54.2)
Female	435 (45.8)
NBW (g), mean ± SD	3,354 ± 470

*Abbreviations:* BMI, body mass index; NBW, neonatal birth weight; SD, standard deviation.

**Fig.1:**
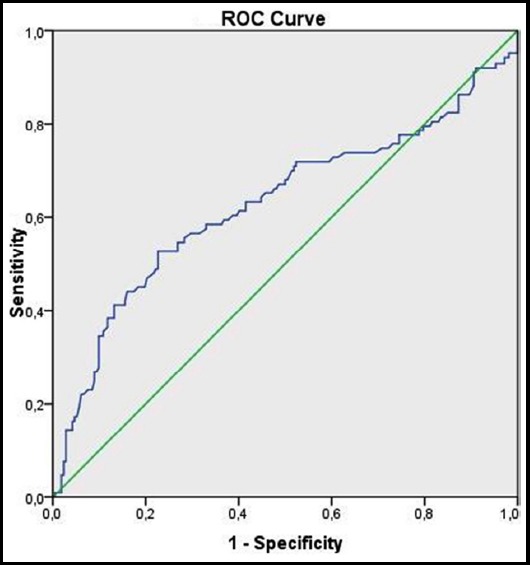
Receiver operating characteristic (ROC) curve of neonatal birth weight for determining sonographic fetal weight estimation accuracy (i.e., success vs. failure) (area under the curve: 0.63, standard error: 0.02).

Except for the mean NBW, fetal presentation, and parity, the groups did not differ according to the investigated parameters (Table-II). Primiparity and cephalic presentation of the fetus were significantly more common in the failure group than in the success group (55.9% [175/313] *vs*. 44.8% [285/636] and 98.1% [307/313] *vs*. 95.2% [606/636]; *p* = 0.001 and *p* = 0.030, respectively). Conversely, the mean NBW was significantly lower in the failure group than in the success group (3,250 ± 565 *vs*. 3,404 ± 410 g, respectively; *p* = 0.001). There was a significant negative linear correlation between the APE and NBW (Pearson’s correlation coefficient, *p* = 0.001). Receiver operating characteristic curve analysis revealed that the optimal cutoff value of NBW for discriminating between the groups was 3,300 g. The sensitivity was 60.0% and the specificity was 61.0% (Fig.1).

**Table-II:** Maternal, fetal sonographic, and neonatal characteristics according to sonographic fetal weight estimation accuracy (i.e., success *vs.* failure).

Characteristic	Success	Failure	*p*-value

	(*n* = 636, 67.0%)	(*n* = 313, 33.0%)	
*Maternal*			
Age (years), mean ± SD	28.2 ± 5.4	27.9 ± 5.4	0.570
*Parity, n (%)*			
Primiparity	285 (30.0)	175 (18.4)	0.001
Multiparity	351 (37.0)	138 (14.6)	
Gestational age (weeks), mean ± SD	39.1 ± 1.3	39.0 ± 1.2	0.330
Maternal height (cm), mean ± SD	161.8 ± 5.5	161.5 ± 4.8	0.350
Maternal pregestational weight (kg), mean ± SD	64.8 ± 12.6	65.3 ± 12.0	0.570
BMI (kg/m^2^), mean ± SD	29.1 ± 4.9	29.1 ± 4.5	0.950
Weight gain during pregnancy (kg), mean ± SD	11.5 ± 4.8	10.9 ± 3.9	0.060
*Labor stage, n (%)*			
Active phase	114 (12.0)	57 (6.0)	0.930
Latent phase	522 (55.0)	256 (27.0)
Fetal sonographic			
Estimated fetal weight (g), mean ± SD	3,415 ± 360	3,460 ± 360	0.070
Amniotic fluid volume (mL), mean ± SD	105 ± 36	103 ± 31	0.640
*Placental localization, n (%)*			
Anterior	180 (18.9)	106 (11.2)	0.080
Other (posterior, fundal, or lateral)	456 (48.1)	207 (21.8)
*Fetal presentation, n (%)*			
Cephalic	606 (63.9)	307 (32.3)	
Non-cephalic (breech or transverse)	30 (3.2)	6 (0.6)	0.030
Neonatal			
*Sex, n (%)*			
Male	348 (36.7)	166 (17.5)	0.620
Female	288 (30.3)	147 (15.5)	
NBW (g), mean ± SD	3,404 ± 410	3,250 ± 565	0.001

*Abbreviations:*BMI, body mass index; NBW, neonatal birth weight; SD, standard deviation.

Finally, binary logistic regression analysis identified primiparity (RR: 1.6, 95.0% CI: 1.2 to 2.1; *p* = 0.010), cephalic presentation of the fetus (RR: 2.8, 95.0% CI: 1.1 to 6.8; *p* = 0.020), and a NBW of <3,300 g (RR: 2.4, 95.0% CI: 1.8 to 3.2; *p* = 0.010) as independent risk factors for SFWE failure.

## DISCUSSION

SFWE is significantly correlated with NBW. Previous reports^6^,^8^,^14^,^21^-^23^ investigating the efficiency of sonography have determined that the mean APE is within a 10.0% range of NBW. However, SFWE accuracy remains unsatisfactory, with failure rates of between 32.0% and 83.0%.^9^,^10^ The overall accuracy rate of SFWE has been reported to be 56.0% in full-term pregnant women.^8^ In this study, the mean APE was 8.2% and the SFWE accuracy rate was 67.0%. These findings were consistent with those of previous reports.^6^,^8^-^10^,^14^,^21^-^23^

It has not yet been established whether factors relating to maternal, fetal sonographic, and neonatal characteristics are responsible for SFWE failure. Our findings support this position. For instance, in contrast to previous studies^11^,^14^-^16^,^18^,^19^, we did not identify high maternal body mass index, low amniotic fluid volume, macrosomia, female fetus, high maternal height, and older age as risk factors for SFWE failure. Moreover, there were no significant associations between maternal antenatal weight, weight gain during pregnancy, placental localization (anterior *vs*. other), and stage of labor (active *vs*. latent phase) and SFWE failure.

In the present study, primiparity and cephalic presentation of the fetus were independent risk factors for SFWE failure, with RRs of 1.6 and 2.8, respectively. In contrast to the present study, no significant associations have been identified between parity and fetal presentation and SFWE failure in recent reports.^19^,^24^ Because no comparable data were presented in these studies, we could not determine the cause of dissimilarity between the findings of these studies and our own.

In the present study, there was, however, a significant negative linear correlation between the mean APE and NBW. A NBW of <3,300 g was identified as an independent risk factor for SFWE failure. Below this value, the RR was 2.4 times greater. In contrast to the present study, Colman et al.^23^ failed to identify any significant associations between the mean APE and SFWE failure and NBW in a study population comparable to ours. The inconsistency between studies may be the result of many unpredictable factors in the study population, such as race, practitioner experience, and whether the characteristics were investigated in our study.

## CONCLUSIONS

In conclusion, although the present study has its strengths (the prospective study design and taking into consideration the technical characteristics) and limitations (single-center study on uncomplicated pregnant women), it revealed a significant association between SFWE and NBW. Conversely, our study showed that SFWE accuracy remains unsatisfactory and is dependent on unpredictable and inconsistent factors.
